# Evaluation of a Porous Bioabsorbable Interbody Mg-Zn Alloy Cage in a Goat Cervical Spine Model

**DOI:** 10.1155/2018/7961509

**Published:** 2018-11-25

**Authors:** Haocheng Xu, Fan Zhang, Hongli Wang, Fang Geng, Minghao Shao, Shun Xu, Xinlei Xia, Xiaosheng Ma, Feizhou Lu, Jianyuan Jiang

**Affiliations:** ^1^Department of Orthopedics, Huashan Hospital, Fudan University, No.12 Wulumuqi Middle Road, Shanghai, China; ^2^Department of Research & Tech, Medtronic Greater China Co., Ltd., Block 11, No. 3000 Long Dong Avenue, Pudong, Shanghai, China

## Abstract

**Purpose:**

Bioabsorbable Mg-based implants were previously assessed due to their intrinsic advantages, but Mg-based cage related research is limited. The specific blood supply and stress of the intervertebral environment can affect the function of Mg-based implants. The objective of this study was to investigate the performance of a bioabsorbable Mg-Zn alloy cage in anterior cervical discectomy and fusion (ACDF) and evaluate the control of degradation of the Mg-Zn cage surface modified by microarc oxidation (MAO) technology containing Si under an intervertebral microenvironment.

**Methods:**

Twenty-four goats were divided into four groups according to the experimental period and all underwent ACDF at C2-3 and C4-5 with porous Mg-Zn cage covered with a MAO/Si-containing coating in one intervertebral space and with autologous iliac bone in another space. After 3, 6, 12, or 24 weeks after operation, the cervical spine specimens were harvested to evaluate the biocompatibility, fusion status, and degradation conditions using blood analysis, radiology, biomechanical testing, histology, and micro-CT.

**Results:**

The Mg-Zn cages showed ideal biocompatibility and biomechanical characterization; however, the fusion state, as evaluated with radiology and histology, was not acceptable. Modified by the MAO/Si-containing coating, the degradation rate of the Mg-Zn cages was controllable but slower than expected.

**Conclusion:**

MAO/Si-containing coating Mg-Zn alloy cages demonstrated excessive control of degradation and fusion failure after 24 weeks postoperatively. We conclude that further studies should be designed to improve the using of Mg-based materials at the intervertebral space.

## 1. Introduction

Anterior cervical discectomy and fusion (ACDF) was first described in 1958 using a graft of autogenous bone from the iliac crest [1, 2]. As the gold standard for intervertebral fusion, iliac autograft demonstrated a series of complications such as infection, fractures, and prolonged pain at the donor site [3, 4]. As an alternative, fusion cages have been widely used, and most are made of nondegradable materials, such as PEEK and carbon fiber reinforced polymers. However, bioabsorbable implants can effectively avoid long-term complications, which include stress shielding effects, implant shifts, and foreign body reactions. Additionally, complete intervertebral bony fusion is accomplished as the degradation space can be continuous replaced by osteosis. Among the types of bioabsorbable materials, magnesium (Mg) is considered a potential orthopedic material due to its favorable characteristics, such as biocompatibility, similar mechanical properties to natural bone, osteoconductive activity, and radiolucency [5, 6] (Table 1).

Despite the tremendous advantage of biodegradable Mg-based materials, the major limitations for orthopedic applications is the loss of mechanical integrity, gas accumulation, and local microenvironmental changes in the corrosion process occurring at a fast rate. Because of these drawbacks, strategies such as alloying and surface modifications were proposed to tailor the control of degradation with satisfying results [5]. However, the majority of successful Mg alloy applications were focused on screws and plates, and the only application of Mg alloy as fusion cages was not successful [7]. This may be because in contrast with the long bone, the intervertebral space shows a specific blood supply environment after ACDF and a unique stress stimulation environment, both of which impact the cage corrosion rate [8, 9]. Therefore, it is imperative to explore the appearance of Mg-based cages.

We developed a newly designed porous Mg-Zinc (Zn) alloy cage treated with a microarc oxidation (MAO)/silicon- (Si-) containing coating as the experimental implant based on previous research conclusions from Mg-based materials applied in long bones. Both of the addition of Zn and the MAO/Si-containing coatings can reduce the corrosion rate of Mg-based scaffolds [10–12]. Furthermore, because porosity can benefit the material by increasing biological integration and by adjusting the mechanical properties to comply with the natural bone system [13], so we also introduce a porous design into the production of the cage.

The aim of this study was to investigate the performance of this bioabsorbable porous Mg-Zn alloy cage in ACDF and evaluate the control of degradation of the Mg-Zn cage surface modified by a MAO/Si-containing coating.

## 2. Materials and Methods

### 2.1. Implant

The addition of Zn can affect the corrosion of Mg alloys by the grain refinement and the formation of secondary phases [10, 11]. However, Song et al. [14] indicated that excessive Zn is detrimental to the corrosion resistance of Mg-Zn alloys and the optimal content of Zn in Mg-Zn alloys should be less than 5.0%. At the same time, the mechanical properties of Mg alloys can also be influenced by the addition of Zn. More than 3% addition of Zn to Mg will improve the strength [15] and the Zn content should be limited to 4% to achieve the maximum yield strength and elongation [16]. As a result, the experimental cage was manufactured from an Mg-Zn alloy with the Zn content of 3.5%-4.5% (Table 2).

**Figure 1:**
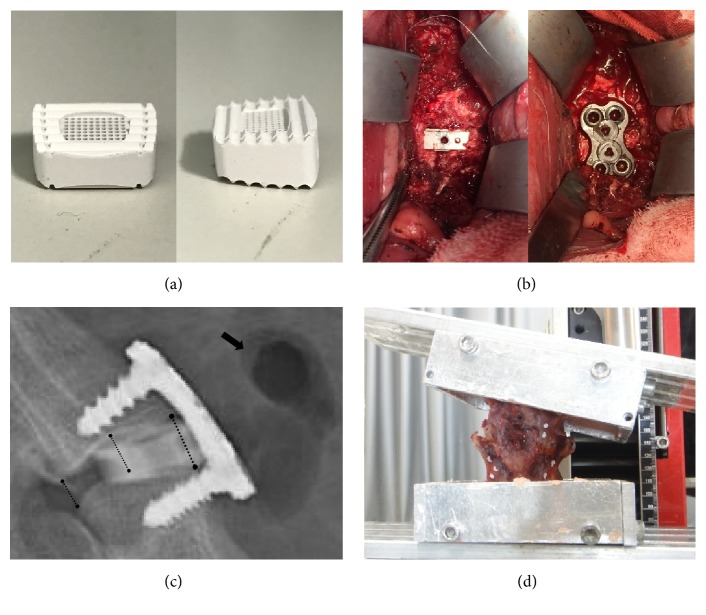
The construction of the goat cervical spine model. (a) The front and the lateral view of the MAO/Si-containing coated Mg-Zn alloy cage. The size was 12 mm*∗*10 mm*∗*4.5-6 mm with a 7-degree wedge angle. The porous structure was designed in the central area of 5*∗*6 mm. (b) The operating field after cage implantation and titanium plate fixation. (c) The sketch map of the measurement of disc space height (DSH) in a cage segment (6 weeks). Three black lines indicate the measured height of the anterior, midpoint, and posterior disc space, and the black arrow indicates gas accumulation in front of the disc space. (d) Stiffness measurement by a nondestructive method using a nonconstrained testing apparatus.

The cage was fabricated in a rectangular design with the specification of 12 mm*∗*10 mm*∗*4.5-6 mm and a 7-degree wedge angle (Figure 1(a)). According to our previous results [17], the porosity of 45% will maximize the physicochemical characteristics and biocompatibility of the cage, so a porous structure was designed in the central area of 5*∗*6 mm with a porosity of 45% and pore size of 400 *μ*m.

After the fabrication of skeleton, the cage was modified by a MAO/Si-containing coating as previously reported [12, 18]. Briefly, the skeleton was treated with silicate-based electrolyte containing Na_2_SiO_3_·9H_2_O (10 g/L), KOH (1 g/L), and KF·2H_2_O (8 g/L) for 10 minutes with a positive voltage of 460 V, pulse frequency of 600 Hz, and positive/negative duty cycles of 30%/20%.

Tested in a universal tester, the cage satisfied the requirements for intervertebral implantation with an elastic modulus of 42.64 ± 2.11 GPa and compressive yield strength of 311.74 ± 14.20 MPa.

### 2.2. Animal Work

The animal study was approved by the Institutional Animal Care and Use Committee (IACUC) at the Department of Laboratory Animal Science, Fudan University (accreditation number 2016-1053-A357). All experimental procedures were described in our previous publication [19].

The goat has been regarded as an ideal experimental model for cervical spine fusion due to the similarities between the goat and human cervical spine [20]. Especially, C2-3 level has the highest similarity to human anatomy and is the most appropriate level for animal testing [21]. A reserved segment between two surgical segments is needed for mechanical analysis, so we underwent ACDF at C2-3 and C4-5.

Twenty-four healthy goats (age: 2-3 yr) were divided into four groups (6 animals per group) based on the experimental period of 3, 6, 12, and 24 weeks and underwent ACDF at C2-3 and C4-5 with a porous Mg-Zn cage in one intervertebral space and with autologous iliac bone in another followed by stabilization with titanium plate fixation, which can provide stability for fusion and minimize the impact between two operation segments (Figure 1(b)). Because autogenous bone from the iliac crest has been the “gold standard” graft material and demonstrated an outstanding fusion rate, we chose autogenous bone as the control. There was an even distribution of the 2 types of implants in the two segments. After 3, 6, 12, or 24 weeks postoperatively, six animals were euthanized and the C2-3 and C4-5 motion segments were harvested and stored at -20°C for further investigation.

### 2.3. Radiographical Analysis

Lateral radiographs were performed after implantation and before sacrifice to determine whether any gas accumulation or intervertebral space subsidence occurred. The disc space height (DSH) was defined as the average of the measured height of the anterior, midpoint, and posterior disc space (Figure 1(c)). A high-resolution CT scan was conducted at the end of the observation period to evaluate the interbody fusion. With a sagittal reconstruction of the CT images, the interbody fusion could be evaluated based on a scoring system reported by Goldschlager et al. [22]. The criteria for CT scoring were as follows: 0, no new bone formation; 1, new bone formation but not continuous between C2/3 and C4/5; 2, continuous bridging new bone but comprises <30% of fusion area; and 3, continuous bridging new bone but comprises >30% of fusion area.

All the CT scans were performed using a GE LightSpeed 16 Slice CT Scanner (GE Healthcare, Milwaukee, WI, USA) with the 0.63 mm slice thickness and 0.63 mm interval.

### 2.4. Biocompatibility Analysis

Blood samples were collected at the start and end of the observation period. Parameters, such as Mg ion concentration, Zn ion concentration, blood urea nitrogen (BUN), and serum creatinine (CREA), were tested to determine the biocompatibility of Mg-Zn alloy.

### 2.5. Mechanical Analysis

After the musculature and anterior titanium plate were removed, the monosegment specimens were mounted with polymethylmethacrylate. The measurement of stiffness was performed using a nondestructive method with a nonconstrained testing apparatus described elsewhere in detail [20]. Pure moments were applied in three anatomical axes (flexion/extension, lateral bending left/right, and axial rotation left/right) under the limitation of a maximum moment (± 2 Nm) and a maximum angle (± 10°). Markers were attached to the vertebral bodies in a noncollinear arrangement. In this way, the range of motion (ROM) could be evaluated using two cameras and recorded by a computerized motion analysis system (Figure 1(d)). During the tests, specimens were kept moist with 0.9% isotonic saline solution in spray bottle. The slope of the linear region in the load displacement curve was calculated and defined as the specimen stiffness.

### 2.6. Degradation Analysis

Micro-CT was used to quantitate cage degradation. Based on the different density values between the Mg-Zn alloy cage and surrounding tissue, the volume of the remaining cage was calculated from the 3D reconstruction. The degradation rate was equal to the volumetric percentage loss of the cage at the 4 defined time intervals.

### 2.7. Histological Analysis

After fixing with paraformaldehyde, dehydration by acetone, and embedding in methylmethacrylate, the specimens were cut into undecalcified hard tissue sections using the cutting-grinding technique. For this purpose, the blocks were cut with a diamond band saw (EXAKT 300 CP, Norderstedt, Germany) into 300-*μ*m-thick slices. Then, these slices were ground with a plate grinder (EXAKT 400 CS, Norderstedt, Germany) to a thickness of 20 *μ*m. Sagittal sections in the midline of the monosegments were obtained. Alizarin red staining and toluidine blue staining were used, respectively, to detect calcium in the mineralized bone parts next to the implant and the degree of osteoblast activity.

### 2.8. Statistical Analysis

Statistical analyses were performed using SPSS12.0 (SPSS, Chicago, IL, USA). A paired t-test was used to compare the change of the mean DSH and hematological parameters. Statistical analyses of the stiffness and CT fusion scores were performed using independent samples t-tests. P < 0.05 was defined as statistically significant.

## 3. Results

A total of 25 goats underwent surgical treatment, 1 of which died due to improper fasting before anesthesia; the remaining goats were included in the study without other general complications.

### 3.1. Biocompatibility Results

**Figure 2:**
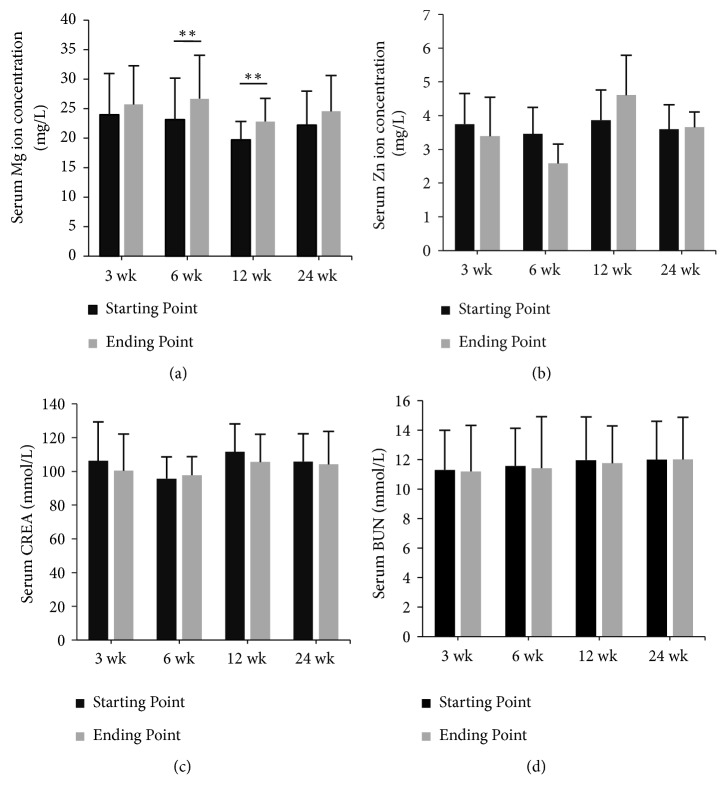
Changes of serum biochemical parameters after treatment with the Mg-Zn alloy. (a) The serum Mg ion concentration increased within 6 weeks and then steadily decreased. The changes of the Mg ion concentration at 6 weeks and 12 weeks were statistically significant (P = 0.0062 and P = 0.0044, respectively). (b), (c), and (d) Significant changes were not observed in the levels of the serum Zn ion concentration, CREA, and BUN (all P > 0.05). The average baseline of the 4 above parameters for all 24 goats was 22.25 ± 5.77 mg/L, 3.66 ± 0.79 mg/L, 11.70 ± 2.53 mmol/L, and 104.82 ± 17.50 *μ*mol/L, respectively.

The biocompatibility results showed that the serum Mg ion concentration increased slightly within 12 weeks postoperatively and steadily declined afterwards. The serum Mg ion concentration increased by 1.76mg/L (7.3%) in 3 weeks postoperatively, 3.54 mg/L (15.3%) in 6 weeks, 3.08 mg/L (15.6%) in 12 weeks, and 2.33mg/L (10.5%) in 24 weeks. No complications associated with hypermagnesemia were found. However, the changes of the serum Zn ion concentration, BUN, and CREA were not statistically significant (Figure 2).

### 3.2. Radiographical Results

A total of 33.3% of the goats had a small amount of gas accumulation in front of the disc space implanted with the Mg-Zn cage (Figure 1(c)), particularly in the 3-week group (5/6) and 6-week group (3/6), but not after 12 weeks. After 6 weeks postoperatively, subsidence of the intervertebral space was observed in the segments with the autogenous iliac graft. The DSH decreased from 4.083 mm to 3.542 mm in 12-week group and from 4.283 mm to 3.533 mm in 24-week group with a subsidence rate of 13.3% and 17.5%, respectively. In the segments with the Mg-Zn cage, the DSH after implantation (5.78 ± 0.26 mm) was significantly higher than that of the segments with autogenous bone (4.51 ± 0.19 mm, p = 0.0003). Meanwhile, there was no significant subsidence in the segments with the Mg-Zn cage (Table 3). Similar to previous studies [7], a bone bridge, which was anterior to the fixation, was formed by osteophytes, particularly after 12 weeks. Because evaluation of the fusion status on lateral radiographs was not accurate [7, 23], we analyzed it on CT scan and using histology.

### 3.3. Biomechanical Results

The stiffness of the segments demonstrated bilaterally symmetrical results as the stiffness in lateral bending (left) and axial rotation (left) was similar to lateral bending (right) and axial rotation (right) in almost groups. Besides, the stiffness in flexion is usually higher than that in extension. With time, a growing stiffness was observed in all segments that increased to different extents, and the segments with bone grafts demonstrated better stability. After 24 weeks, the calculated stiffness for the bone-implanted segments was more than twice as the cage-implants segments with a significantly differences in almost all anatomic axes (Table 4). In addition, significant differences also appeared in the 6-week axial rotation (right), 12-week flexion, 12-week lateral bending (left), and 12-week axial rotation (left). The above findings indirectly suggested that the Mg-Zn cage was related to a poor fusion status.

### 3.4. Degradation Results

**Figure 3:**
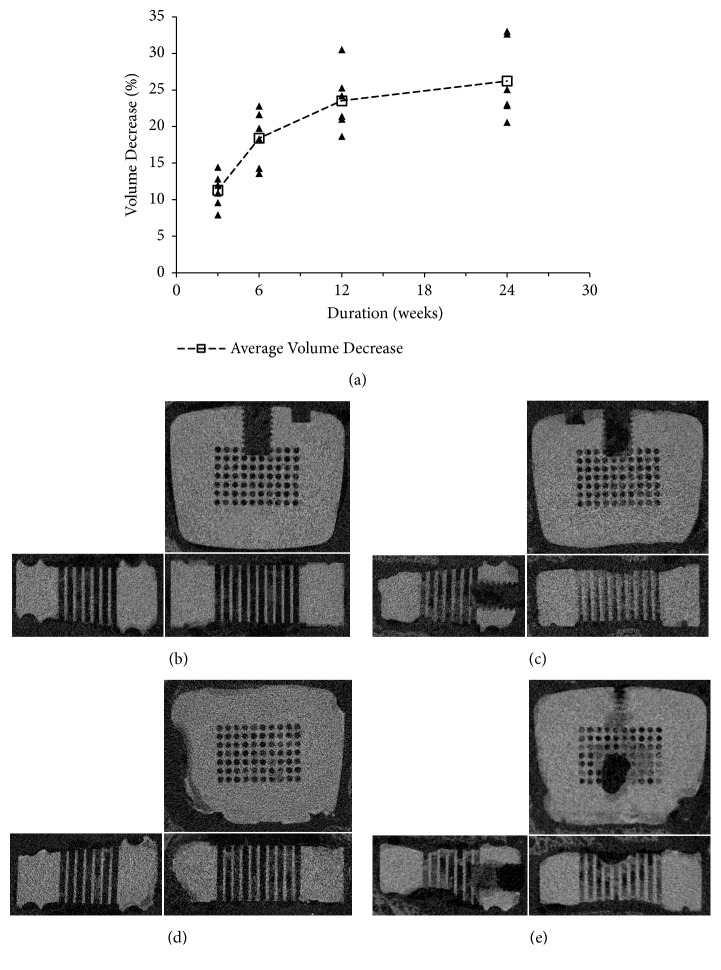
Volume decrease of the cages during degradation. (a) The degradation rate was relatively fast within the first 6 weeks and gradually slower during the following period. (b), (c), (d), and (e) The morphology of MAO/Si-containing coating Mg-Zn alloy cage on micro-CT at 3, 6, 12, and 24 weeks, respectively.

The degradation rate of the cages was relatively rapid within the first 6 weeks and gradually became steady during the subsequent period. In detail, the average volume decrease of the cages was 11.29 ± 2.32 at 3 weeks, 18.42 ± 3.8 at 6 weeks, 23.53 ± 4.17 at 12 weeks, and 26.23 ± 5.33 at 24 weeks (Figure 3).

### 3.5. Fusion Results

**Figure 4:**
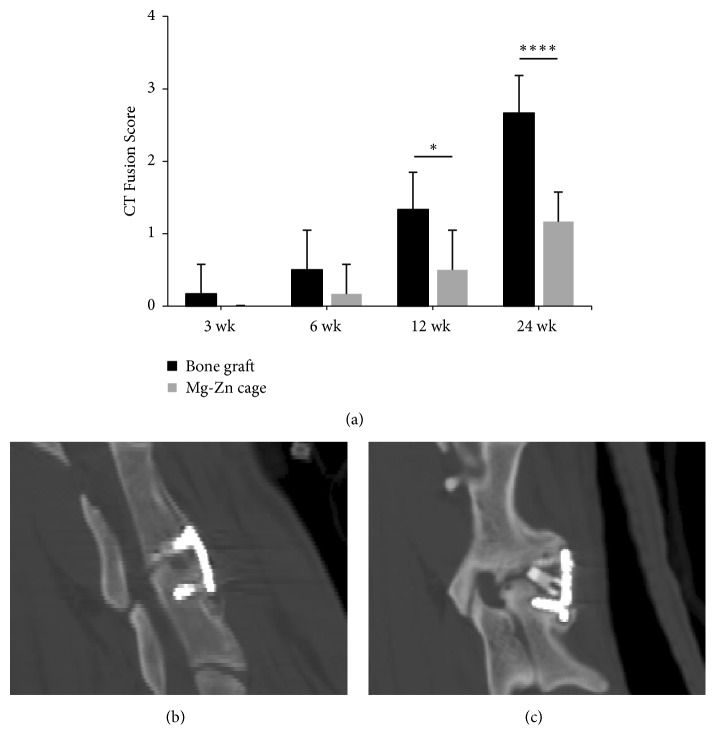
CT fusion scores of the segments treated with two types of implants. (a) The fusion score of segments with a bone graft increased over time (P < 0.0001) and was significantly higher than the segments with a cage graft at 12 weeks and 24 weeks (p = 0.0158 and p = 0.001, respectively). No statistical difference was observed in the degree of fusion at 24 weeks in the segments with a cage, indicating unsuccessful bony fusion between the vertebral bodies. (b), (c) The fusion state of the segment with a bone graft is significantly superior to the segment with a cage graft at 24 weeks on sagittal reconstruction of the CT images.

The analysis of interbody fusion was conducted using CT 3D reconstruction. During the observation period, the fusion score of the segments with both cage and bone graft increased over time (P = 0.0003 and P < 0.0001, respectively). However, the fusion score of segments with a bone was significantly higher than the segments with a cage at 12 weeks and 24 weeks (P = 0.0219 and P = 0.0002, respectively) (Figure 4(a)). After 24 weeks, continuous new bone tissue between the endplates in the segments with bone graft could be found in the CT 2D reconstruction image (Figure 4(b)), whereas there was no obvious progress in the fusion state in the segments with a cage (Figure 4(c)).

**Figure 5:**
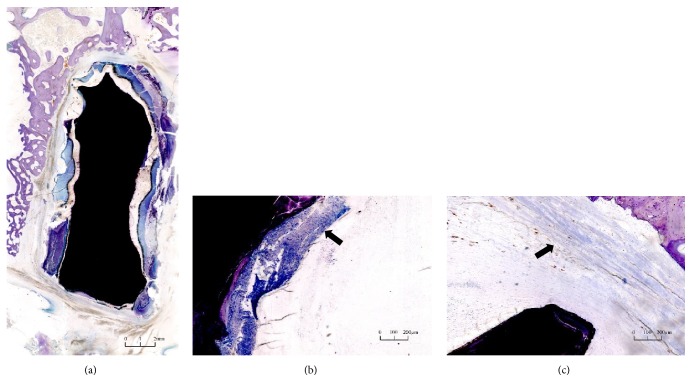
Histological sections after 24 weeks after operation. (a) Osteoid tissue was not observed to grow into the cage and there was a gap between the bone tissue and cage. (b) Infiltrating inflammatory cells in the gap. (c) Hyperplastic fibrous tissue capsule in the gap.

In segments with a bone graft, bone trabeculae, some of which formed continuous bone bridges, were bulky and dense between the endplate and the implanted autogenetic iliac bone after 24 weeks. However, similar to the CT results, the fusion condition in the segments with a cage was not satisfactory. Osteoid tissue was not observed to grow into a cage after 24 weeks, and a gap appeared between the bone tissue and cage (Figure 5(a)). Material abscission components, embedding matrix, infiltrating inflammatory cells, and hyperplastic fibrous tissue capsules were observed in the gap (Figures 5(b) and 5(c)). There was no obvious osteonecrosis, granuloma, and bone dissolution around the cages.

## 4. Discussion

As shown by several studies, the biocompatibility of Mg-based materials performed well in vivo. During the corrosion process, the release rate of Mg ions would not exceed the endurance limitation of the human body, and the degradation products were excreted in the urine as soluble and nontoxic oxides [24]. A slight increase in the serum Mg ion concentration was observed in our goat models. An excessive serum Mg concentration is associated with muscular paralysis, hypotension, and respiratory distress [25]; however, no related complications were seen in the goat model, and both BUN and CREA were maintained at normal levels, which indicates that the serum Mg concentration was in safe ranges, and no evidence of renal insufficiency was detected.

Mg-based materials had excellent mechanical properties, balancing between autogenous bone and metal materials, thereby reducing the probability of intervertebral space subsidence and stress shielding effects [6]. In our initial study, the elastic modulus and yield strength of the Mg-Zn cages were 42.64 ± 2.11 GPa and 311.74 ± 14.20 MPa, respectively. Although the elastic modulus of the cage is twice the maximum value reported for natural bone (15-25 GPa), this result is superior to other cage materials and matched that of natural bone well. Additionally, the biomechanical characteristics of the Mg-Zn cages also performed well in radiographical analysis as the degree of intervertebral space subsidence was clearly lower than the autologous bone.

A degradable Mg-based material was introduced in orthopedics in the early 20^th^ century [26]. Despite the promise of Mg-based materials that have tremendous advantages, the low corrosion resistance and secondary complications severely limit their use. So it is important to comprehend the corrosion mechanism of Mg and its alloys and tailor scaffold degradation in a manner which is suitable for an in vivo environment.

The corrosion of Mg-based scaffold is slow in the typical atmosphere owing to the formation of an oxide film of magnesium hydroxide (Mg(OH)_2_) and hydrogen gas. Mg(OH)_2_ is not soluble and can protect the scaffold away from further degradation. However, chloride ions in in vivo or in vitro surrounding environment may react with the films to form highly soluble magnesium chloride (MgCl_2_) and hydrogen gas, which may accelerate the degradation [27].

The basic conditions that determine the corrosion rate are alloy compound and environment around implant. Previous studies demonstrated that alloying and surface modification tailor the corrosion resistance of Mg-based materials [5], and both the Mg-Zn alloy and MAO/Si-containing coating showed excellent performance with numerous strategies [10–12]; therefore, a MAO/Si-containing coated Mg-Zn alloy cage was selected.

The degradation rate of Mg also depends on surrounding environment. For example, the in vitro corrosion rate varies with experimental methods, media of corrosion test, experimental temperature, pH around material, and the use of dynamic condition [28]. Furthermore, the difference in water contents, blood flow, and chloride contents may affect the in vivo degradation rate [29]. As a result, the in vivo corrosion rate is on average 1-5 times higher than the corrosion rate obtained in vitro [29].

Although Mg-based screws achieved approval for clinical applications in March 2013, the corrosion behavior of Mg-based cages remained poorly understood. The degradation rate is different in various implantation sites. First, several studied demonstrated that the degradation rate of Mg-based scaffolds is related to the blood supply [30, 31]. The amount of blood flow can affect the removal of degradation product and prevent the formation of protective layer on alloy surface, so the Mg-based scaffold usually had higher degradation rate in cancellous bone than in cortical bone. The blood supply of the intervertebral space after ACDF is different from that of long bone, which is proposed to be one of the most important factors influencing the corrosion rate of Mg-based cages [32]. Second, potential biodegradable scaffolds would be exposed to different stress environment in vivo, depending on the implantation site [6]. The difference in mechanical stress, including fluid shear stress and compression between Mg-based cages and screws also determines the unique characteristics of the resulting corrosion rate [8, 33].

As a result, MAO/Si-containing coated Mg-Zn alloy cages manifest a relatively rapid degradation rate within the first 6 weeks and gradually become steady during the subsequent period, with a volumetric percentage loss of 26.23% at 24 weeks. In addition, obvious gas accumulation was found at 3 and 6 weeks, which was consistent with the volume changing rate. An acceptable degradation rate is beneficial in orthopedic applications.

As previously discussed, the first application of an Mg alloy as a fusion cage in an ovine model was unsuccessful [7]. Unfortunately, our research yielded a similar result, even with a newly designed porous Mg-Zn cage covered with a MAO/Si-containing coating. Compared with segments using bone graft, all biomechanical findings, CT fusion scores, and histological results revealed an inferior fusion state in segments with a cage. Even after 24 weeks, there was no evidence of bony fusion in the disc space around or through the cage. As interbody fusion failure should be much lower than the nonunion rate of long bone, it will be interesting to determine why Mg-based implants cannot improve osteogenesis at the intervertebral space [34–36].

We attributed this phenomenon to the hindering of new bone ingrowth by Mg accumulation. Despite the osteoinduction of Mg ions [37], excessive Mg accumulation could result in a severe foreign body response, gas accumulation, tissue irritation, abnormal calcium precipitation [31, 38], and finally impaired osteogenesis. The intervertebral space has a special surrounding blood supply and stress stimulation as stated above; therefore, an Mg-based cage may present unique corrosion behavior and excessive intervertebral Mg accumulation. This can facilitate an understanding of the association between cage degradation and osteogenesis.

Despite the poor behavior of Mg-based alloys in the intervertebral space in this study, we propose that they remain a promising material for cages due to their previous impressive results as other orthopedics implants. Thus, we should evaluate further component modifications, explore different surface modifications, and investigate the unique ion distribution in the intervertebral space.

## 5. Conclusions

MAO/Si-containing coating Mg-Zn alloy cages demonstrated showed an ideal biocompatibility without any internal environment disorder or renal insufficiency happening during the experiment. The disc space height of segments with cage remains normal after 24 weeks indicates that Mg-based cages had sufficient mechanical strength for intervertebral application. In addition, the degradation rate of the Mg-Zn cages was controllable under the MAO/Si-containing surface modification. However, the fusion state, as evaluated with radiology and histology, was not acceptable. The hindering of new bone ingrowth by Mg accumulation might be the reason of fusion failure. As a result, further studies should be designed to improve the using of Mg-based materials at the intervertebral space and solve the bony growth inhibition caused by Mg accumulation.

## Figures and Tables

**Table 1:** Comparison of magnesium and two common fusion cage materials.

	Titanium	PEEK	Magnesium
Elastic Modulus	High	Close to bone	Close to bone
Subsidence Rate	High	Low	Low
Compressive Strength	High	Close to bone	Close to bone
Stability	Excellent	Well	Well
Radiolucent	No	Yes	Yes
MRI Compatible	No	Yes	Yes
Osteointegration	Yes	Yes	Yes
Degradable	No	No	No
Biocompatibility	Normal	Well	Excellent

**Table 2:** Chemical composition of the Mg-Zn alloy.

Material	Chemical composition (wt.%)								
	Zn	Ni	Zr	Fe	Si	Mn	Cu	Al	Mg
Mg-Zn	3.5-4.5	≤0.02	≤0.02	≤0.01	≤0.01	≤0.05	≤0.01	≤0.01	Balance

**Table 3:** Collapse of the DSH in segments with two implant types.

		**3 wk**	**6 wk**	**12 wk**	**24 wk**
Bone	ΔDSH	0.23 ± 0.28	**0.41 ± 0.35**	**0.54 ± 0.48**	**0.75 ± 0.45**
	P	0.1057	**0.0349**	**0.0397**	**0.0098**
Cage	ΔDSH	0.00 **± **0.32	0.51 **± **0.72	**0.51 **±** 0.35**	0.07 **± **0.81
	P	0.9806	0.1448	**0.0151**	0.8364

ΔDSH refers to the variation of DSH and positive value indicates the decrease of DSH. Statistically significant differences during period (*α* = 0.05) are marked by boldface text (paired t-test).

**Table 4:** Stiffness of the segments treated with two types of implants (bone graft versus cage) in the 3 anatomical axes.

		**3 wk**	**6 wk**	**12 wk**	**24 wk**
Flexion (N·m/mm)	Cage	0.52 ± 0.21	0.68 ± 0.25	**0.70 **±** 0.26**_ _^**∗∗∗**^	**1.06 ± 0.28**_ _^**∗∗∗**^
	Bone	0.58 ± 0.26	1.01 ± 0.48	**1.40 ± 0.24**_ _^**∗∗∗**^	**2.42 ± 0.59**_ _^**∗∗∗**^
Extension (N·m/mm)	Cage	0.35 ± 0.10	0.54 ± 0.18	0.62 ± 0.30	**0.79 ± 0.14**_ _^**∗∗∗**^
	Bone	0.43 ± 0.15	0.51 ± 0.27	1.06 ± 0.39	**1.69 ± 0.42**_ _^**∗∗∗**^
Lateral bending (left) (N·m/mm)	Cage	0.30 ± 0.04	0.45 ± 0.27	**0.62 ± 0.18**_ _^**∗∗**^	**0.77 ± 0.37**_ _^**∗∗∗**^
	Bone	0.26 ± 0.07	0.55 ± 0.18	**1.23 ± 0.29**_ _^**∗∗**^	**2.31 ± 0.60**_ _^**∗∗∗**^
Lateral bending (right) (N·m/mm)	Cage	0.34 ± 0.12	0.46 ± 0.23	0.77 ± 0.24	**0.71 ± 0.29**_ _^**∗∗∗**^
	Bone	0.45 ± 0.06	0.64 ± 0.21	1.10 ± 0.34	**1.94 ± 0.55**_ _^**∗∗∗**^
Axial rotation (left) (N·m/°)	Cage	0.22 ± 0.07	0.39 ± 0.17	**0.44 ± 0.23**_ _^**∗**^	**0.48 ± 0.14**_ _^**∗∗∗∗**^
	Bone	0.30 ± 0.12	0.53 ± 0.25	**0.71 ± 0.13**_ _^**∗**^	**1.18 ± 0.13**_ _^**∗∗∗∗**^
Axial rotation (right) (N·m/°)	Cage	0.21 ± 0.07	**0.39 ± 0.18**_ _^**∗**^	0.57 ± 0.17	**0.51 ± 0.15**_ _^**∗∗∗**^
	Bone	0.38 ± 0.19	**0.74 ± 0.30**_ _^**∗**^	0.69 ± 0.23	**1.09 ± 0.20**_ _^**∗∗∗**^


Statistically significant differences between two types of implants (*α* = 0.05) are marked by boldface text (*∗*: p < 0.05; *∗∗*: p < 0.01; *∗∗∗*: p < 0.001; *∗∗∗∗*: p < 0.0001, independent-sample t-test).

## Data Availability

The data used to support the findings of this study are available from the corresponding author upon request.
